# The College of Nursing

**Published:** 1918-06-01

**Authors:** 


### THE COLLEGE OF NURSING (Limited by Guarantee).

### Sheffield Whole-heartedly Forms a Local Centre.

An enthusiastic meeting was held at the Town Hall, Sheffield, under the Presidency of the Lord Mayor, Mr. Alderman A. Cattell, on the 24th ult. There was a large representative attendance, which included two hundred nurses at least. The Honourable Sir Arthur Stanley, G.B.E., M.P., the Chairman of the College of Nursing, was greeted by a welcome so warm and enthusiastic that it gave him an absolute assurance that Sheffield was with him in the enterprise and meant to do its part to make the College Centre a mainstay and help to every member of the nursing profession within the area of its influence.

### The Citizens' Pleasure.

Sheffield is a city full of interest, and since the advent of the late Mr. John Marshall, who for some forty years was actively associated with the administration of the Royal Infirmary there, having acted as its Chairman from 1890 until his death some fifteen years later, it gradually became one of the noted hospitals outside the metropolis. In The Hospital of October 5, 1912, we published the history and wise traditions of the Royal Infirmary, which were, and have been, well maintained by its managers and staff, and have given it a first claim to the support of the citizens of Sheffield. Sheffield contains other interesting hospitals, and altogether it may be said of this city that it has done much to keep well in touch with modern hospital developments. We are assured its citizens will make it their pleasure, as it will also be regarded as their business and privilege, to secure that the Sheffield Centre of the College of Nursing (Limited by Guarantee) shall be second to none in its usefulness, and the blessings and comforts and help it will afford to trained nurses, directly through its agency, and indirectly as the means of bringing every self-supporting and independent nurse speedily to become a member of the College.

### Sheffield Centre Warmly Welcomed.

The meeting on the 24th ult. was enthusiastic throughout, and if confirmation were needed as to what we have already claimed for Sheffield, it might be found in the fact that the Sheffield Centre will be the eighth local centre of the College of Nursing, those already established being at Liverpool, Manchester, Birmingham, Leeds, Leicester, Bristol, and Plymouth. Amongst the points made by Sir Arthur Stanley in his speech, in reply to questions, were that he believed the College Register would form the basis of the State Register; that the College had never drawn any distinction between the nurses trained in a general hospital and a Poor-Law hospital; that nurses' salaries at present were not satisfactory and must be materially increased. The reception given to him and the College movement by the large audience present cheered him beyond words and evidenced the progress which the College of Nursing had made and was making by leaps and bounds throughout Great Britain. Sir William Ellis, Master Cutler, proposed, the Bishop of Sheffield seconded, and Major Finch supported the resolution approving that a local centre of the College of Nursing be formed for Sheffield, which was unanimously carried with applause.

**Sir Arthur Stanley, G.B.E.,** stated that the College was founded to organise the nursing profession and to secure State registration for trained nurses. The nursing sisterhood asked them to form a College of Nursing, and they were only too anxious to do what they could so that not only would nurses be recognised individually but as a society. Everyone wanted to manage their own affairs nowadays, and nurses had been very far from doing anything of the kind. In the College of Nursing they had endeavoured to evolve a constitution upon a democratic basis with the fullest possible representation of the trained nurse. The College of Nursing was working to offer greater advantages in the educational, representative, developmental and professional senses than could be met with elsewhere. Every trained nurse could become a registered member of the College of Nursing, and if qualified, have her name placed upon its register at a total and inclusive cost of one guinea paid by herself. There were no other payments, and this guinea made her a life member of the College, entitled to receive and enjoy all its privileges, and to have behind her the full influence of the College and all connected with it. At present the values attaching to nurses' certificates were extraordinarily different, and they set out to try and standardise the education of nurses, and out of that had grown the College of Nursing. They had for the first time built up a large register of nurses, and hoped that very shortly no representative town would be without its local centre of the College of Nursing. Sheffield's welcome was most encouraging. They would be able to tell the nurses who were working so hard at home and abroad that, when this terrible strain of war was over, and they came back to their ordinary duties, they would find a strongly organised body ready to speak in their name and ready to claim the rights which had for so long been denied them. The will and wish of himself and his colleagues, indeed of all connected with the College of Nursing, was to raise the standard of the nursing profession and to provide that every trained nurse should in confidence turn to the College for help when she found herself in any difficulty, feeling assured that there she had a strong and powerful ally which must materially aid her throughout her professional life and prove at all times a tower of strength.

We are indebted to the *Sheffield Telegraph* for the substance of this report, to which influential and interesting newspaper we have once more to express our obligation.

### Bradford Royal Infirmary Nurses' League.

A meeting of the members of this League was held on May 25 to give an opportunity, as Miss Davies, the president, explained, for members to hear for themselves an account of the work being done by different nursing organisations. Miss Cowlin, organising secretary of the College of Nursing (Limited by Guarantee), was the representative of the College, and Miss Rimmer, of the National Union of Trained Nurses, represented that organisation, which she claimed had provided work for a great many nurses. Miss Rimmer also stated that the National Union of Trained Nurses had first obtained the recognition of nurses' societies on public bodies. Miss Cowlin, in an interesting address, placed the objects of the College before the meeting, dwelling especially on its educational aims. Already the existence of the College had opened the way for improved opportunities and a higher education for its members. Miss Cowlin emphasised the fact that the College was not hostile to any existing organised nurses' society. Since all were working for nursing progress all must have much in common, and it was hoped that at an early date some agreement with regard to a conjoint Bill for the State Registration of Nurses would be arrived at.

